# Demographics of sources of HIV-1 transmission in Zambia: a molecular epidemiology analysis in the HPTN 071 PopART study

**DOI:** 10.1016/S2666-5247(23)00220-3

**Published:** 2024-01

**Authors:** Matthew Hall, Tanya Golubchik, David Bonsall, Lucie Abeler-Dörner, Mohammed Limbada, Barry Kosloff, Ab Schaap, Mariateresa de Cesare, George MacIntyre-Cockett, Newton Otecko, William Probert, Oliver Ratmann, Ana Bulas Cruz, Estelle Piwowar-Manning, David N Burns, Myron S Cohen, Deborah J Donnell, Susan H Eshleman, Musonda Simwinga, Sarah Fidler, Richard Hayes, Helen Ayles, Christophe Fraser, HPTN 071 (PopART) Phylogenetics protocol team, Yaw Agyei, Nulda Beyers, Peter Bock, Virginia Bond, Justin Bwalya, Anne Cori, Anneen Deventer, Rory Dunbar, Wafaa El-Sadr, Lynda Emel, Sian Floyd, Sam Griffith, James Hargreaves, Katharina Hauck, Tanette Headen, Graeme Hoddinott, Anelet James, Karen Jennings, Sarah Kanema, Barry Kosloff, James Kruger, Ramya Kumar, David Macleod, Nozizwe Makola, Nomtha Mandla, Eric Miller, Ayana Moore, Lawrence Mwenge, Heather Noble, Mwelwa Phiri, Michael Pickles, Kalpana Sabapathy, Ephraim Sakala, Rafael Sauter, Kwame Shanaube, Andrew Silumesi, Nirupama Sista, Tim Skalland, Peter Smith, Ranjeeta Thomas, Sten Vermund, Rhonda White, Ethan Wilson, Blia Yang, Krista Yuhas, PANGEA consortium, Rory Bowden, Vincent Calvez, Max Essex, Kate Grabowski, Ravindra Gupta, Joshua Herbeck, Joseph Kagaayi, Pontiano Kaleebu, Jairam Lingappa, Sikhulile Moyo, Vladimir Novitsky, Thumbi Ndung’u, Deenan Pillay, Thomas Quinn, Andrew Rambaut, Janet Seeley, Deogratius Ssemwanga, Frank Tanser, Maria Wawer

**Affiliations:** a Pandemic Sciences Institute and Big Data Institute, Nuffield Department of Medicine, University of Oxford, Oxford, UK; b Sydney Infectious Diseases Institute, School of Medical Sciences, Faculty of Medicine and Health, University of Sydney, Sydney, NSW, Australia; c Wellcome Centre for Human Genetics, Nuffield Department of Medicine, University of Oxford, Oxford, UK; d Zambart, University of Zambia, Lusaka, Zambia; e Department of Clinical Research, London School of Hygiene & Tropical Medicine, London, UK; f Department of Infectious Disease Epidemiology, London School of Hygiene & Tropical Medicine, London, UK; g Department of Mathematics, Imperial College London, London, UK; h Department of Infectious Disease Epidemiology, Imperial College London, London, UK; i Department of Pathology, Johns Hopkins University School of Medicine, Baltimore, MD, USA; j Division of AIDS, National Institute of Allergy and Infectious Diseases, National Institutes of Health, Rockville, MD, USA; k Department of Medicine, University of North Carolina at Chapel Hill, Chapel Hill, NC, USA; l Fred Hutchinson Cancer Research Center, Seattle, WA, USA

## Abstract

**Background:**

In the last decade, universally available antiretroviral therapy (ART) has led to greatly improved health and survival of people living with HIV in sub-Saharan Africa, but new infections continue to appear. The design of effective prevention strategies requires the demographic characterisation of individuals acting as sources of infection, which is the aim of this study.

**Methods:**

Between 2014 and 2018, the HPTN 071 PopART study was conducted to quantify the public health benefits of ART. Viral samples from 7124 study participants in Zambia were deep-sequenced as part of HPTN 071-02 PopART Phylogenetics, an ancillary study. We used these sequences to identify likely transmission pairs. After demographic weighting of the recipients in these pairs to match the overall HIV-positive population, we analysed the demographic characteristics of the sources to better understand transmission in the general population.

**Findings:**

We identified a total of 300 likely transmission pairs. 178 (59·4%) were male to female, with 130 (95% CI 110–150; 43·3%) from males aged 25–40 years. Overall, men transmitted 2·09-fold (2·06–2·29) more infections per capita than women, a ratio peaking at 5·87 (2·78–15·8) in the 35–39 years source age group. 40 (26–57; 13·2%) transmissions linked individuals from different communities in the trial. Of 288 sources with recorded information on drug resistance mutations, 52 (38–69; 18·1%) carried viruses resistant to first-line ART.

**Interpretation:**

HIV-1 transmission in the HPTN 071 study communities comes from a wide range of age and sex groups, and there is no outsized contribution to new infections from importation or drug resistance mutations. Men aged 25–39 years, underserved by current treatment and prevention services, should be prioritised for HIV testing and ART.

**Funding:**

National Institute of Allergy and Infectious Diseases, US President's Emergency Plan for AIDS Relief, International Initiative for Impact Evaluation, Bill & Melinda Gates Foundation, National Institute on Drug Abuse, and National Institute of Mental Health.

## Introduction

The past decade has seen a global transformation in HIV care, with the near universal availability of affordable and effective combination antiretroviral therapy (ART), which durably suppresses viral replication, prevents AIDS, and stops onward transmission of the virus. The discovery that combination ART blocks transmission led to the idea that an effective form of HIV prevention is HIV testing, followed by initiation of ART for infected individuals.^1^

**Research in context**

**Evidence before this study**

We searched PubMed, with no date or language filters, to identify previous quantitative studies investigating the role of age, sex, mobility, and drug resistance mutations (separately or in combination) as drivers of new heterosexual HIV transmissions in sub-Saharan Africa. Observational studies were considered along with those using phylogenetic or mathematical modelling methodologies.

In observational studies, having an older partner or a migrant partner is frequently identified as a risk factor for HIV acquisition, particularly in women, but this is a slightly separate question to the quantification of the overall frequency of these demographics among the sources of new infections. The most recent studies of drug resistance have shown an increasing prevalence, particularly in non-nucleoside reverse transcriptase inhibitor resistance.

The ability to use phylogenetics to investigate HIV transmission is reasonably recent, and the ability to use it to reconstruct who infected whom in transmission pairs is more recent still. One previous, influential study in South Africa posited a key role of men in their 30s in infecting very young women, and being themselves infected by women in their 30s, but that work did not use a methodology that was able to accurately reconstruct direction of transmission. A more recent study in Botswana found similar age distributions for male and female sources of transmission, with the average individual being in their late 30s or early 40s. However, these ages were recorded at the time of study enrolment and do not take into account the time from infection to sampling. The paper also showed that, in that setting, the majority of transmissions were between members of the same community. A number of other phylogenetic studies have been concerned with the very specific dynamics of HIV-1 in the fishing communities of Lake Victoria. No previous phylogenetics work to our knowledge has considered the distribution of drug resistance mutations in sources, and none considered the interaction between source characteristics.

We were unable to find any previous mathematical modelling studies for which the characterisation of sources according to these variables was a major focus.

**Added value of this study**

Our methodology uses a phylogenetic approach to identify likely transmission pairs and the direction of transmission between their members. We find a total of 300 pairs, larger than any previous study. We demonstrate a novel and simple new approach to accounting for potential sampling bias. We also employ a methodology that allows us to estimate the ages of the individuals involved at the time of transmission, rather than that of sampling, countering a key bias in previous approaches. Partly for this reason, our age profiles for sources peak at earlier ages than in previous work, with an average in the early 30s for male sources and mid-20s for females. We fail to confirm the existence of a “renewal cycle” of transmission involving a major contribution from women in older age groups. We also examine the contribution of outside-community transmission and drug resistance mutations, and, for the first time, show that these three characteristics (age and sex, migration, and drug resistance) operate on separate axes and do not cluster together. We use our results to calculate the relative contribution of male to female sources to transmission in age bands, finding that this grows to a peak in the 35–39 years age group in which men are responsible for almost six times as many new infections per person as women.

**Implications of all the available evidence**

The heterosexual HIV epidemic in sub-Saharan Africa appears to be maintained by transmissions from young women and slightly, but not substantially, older men. The contributions of sources transmitting drug-resistant virus, or sources who reside outside a focal community, are not particularly large, and there is no disproportionate contribution from individuals who share any combination of a high-risk age group, a residence outside the community, and drug-resistant virus.

In generalised HIV epidemics, it is tempting to attempt to identify particular demographic groups, of relatively small sizes, for whom intensive targeting of prevention measures will have a major effect on transmission in the general population. The current state of the evidence suggests that this might not be possible, with transmission coming most frequently from younger individuals, disproportionately men, whose demographics are otherwise quite typical of their community. While it might be more difficult and resource-intensive to design universal interventions for the whole population, there can be no shortcuts.

The HPTN 071 (PopART) cluster-randomised trial^2^ evaluated whether a prevention package including universal testing and treatment (UTT) would reduce HIV incidence in communities in Zambia and South Africa. The trial had three arms, with arms A and B testing variations of the UTT package and arm C serving as a control arm; for full details see the trial paper.^2^ HIV prevalence at baseline was between 21% and 22%. The trial reported a 20% reduction of incidence in intervention arms. A further rapid and sustained decrease in new cases is needed to reach the ambitious UNAIDS goals for reduced incidence,^3^ which include 95-95-95 testing and treatment targets within all subpopulations and age groups.

The HPTN 071-02 (PopART) Phylogenetics Study was set up in the Zambian communities of HPTN 071. It aimed to use viral genomes to characterise sources of ongoing transmission, assess the effectiveness of the intervention had it been rolled out nationwide, and identify the most promising policies for prevention in the future.

HIV phylogenies can reconstruct the demographic and spatial history of transmission. In population-level analyses, phylogenetic inference has been used to describe the origin and global spread of HIV,^4^ characterise transmission dynamics in concentrated epidemics,^5^ and, building on studies examining individual transmission events,^6^ analyse transmission in HIV prevention trials.^7^ In sub-Saharan African settings, phylogenetics has been used to look at patterns of clustering^8^ and spatial spread.^9^^,^^10^

Recent methodological innovations in phylogenetics have made it possible, in large studies with high sampling density and after de-identification, to identify probable transmission pairs.^11^ In a sub-Saharan African context, this approach has been successfully used to disentangle patterns of HIV-1 transmission surrounding Ugandan fishing communities on Lake Victoria.^9^^,^^10^ Here, we apply it to HPTN 071-02 data to investigate the generalised epidemic in Zambia. We characterised probable sources of transmission by sex and estimated age at transmission, as well as whether they resided in a different community from their recipient, and if their dominant viral strain was resistant to first-line ART.

## Methods

### Trial participants

HPTN 071-02 was conducted in nine of 12 Zambian PopART communities and included participants from a population cohort (used to monitor incidence in the community) and additional individuals attending health-care facilities. The population cohort enrolled one randomly selected resident aged 15–44 years per randomly selected household, irrespective of HIV status, between Nov 28, 2013, and July 14, 2017, for a total of 20 264 participants in the nine communities. Residents could be visited several times over the course of the trial, with clinical samples being acquired upon the first visit at which they were HIV-positive. At the health-care facilities, individuals were approached and subsequently enrolled if they consented and were HIV-positive individuals aged 18 years or older who were not currently on ART. They were enrolled between July 6, 2016, and June 29, 2018. Demographic data collected included sex, date of birth, and study community. Details can be found in the trial protocol.^12^ Participants gave written consent and the study was approved by the Institutional Review Board of the National Institutes of Health, and the medical ethics committees of the University of Zambia and the London School of Hygiene & Tropical Medicine.

### Genome sequencing

Sequences for viral RNA in each sample were obtained using veSEQ-HIV, a bait-capture based quantitative high-throughput whole-genome deep-sequencing method with a sensitivity of more than 5000 RNA copies per mL for whole genomes.^13^ For further details, see the appendix (p 1).

### Bioinformatic processing

Sequence reads were filtered to remove human and bacterial sequences using Kraken,^14^ and assembled into contigs using SPAdes version 3.10.1.^15^ For every sample, contigs were aligned to 165 representative HIV-1 genomes taken from the 2016 Sequence Compendium from the Los Alamos National Laboratory HIV database to generate a consensus sequence. Individual HIV-1 reads were then mapped onto this consensus. Per-sample HIV-1 genomes were assembled with *shiver* version 1.3.^16^ This generated a custom consensus sequence for each sample; we kept only samples for which at least 50% of consensus positions had non-missing data. Bases in the consensus sequence were not called if the coverage was less than 5 at that position. For depths above this, the majority nucleotide was used, or an ambiguity code in the case of ties. Reads were coordinate-translated by *shiver* to bring them into alignment with the HXB2 reference genome. 910 of 6864 samples were sequenced more than once; for these we combined all reads into a single pool. Subtyping of consensus sequences was performed using REGA.^17^

### Identification of transmission pairs

An HIV-TRACE^18^ analysis with an intentionally generous threshold of 0·04 was performed on the consensus sequences. This was not intended as a rigorous cluster analysis and was instead solely to subdivide the dataset and thus reduce computational time for subsequent phylogenetic reconstruction. Identification of probable transmission pairs was then conducted on the *shiver*-aligned reads in each cluster using *phyloscanner*^11^ (see appendix p 2 for full *phyloscanner* procedure and appendix pp 10–12 for all software settings).

### Estimation of time since infection

HIV-phyloTSI^19^ was used to estimate the infection date of each individual from their within-host phylogenetic data and sampling date. For pairs, this allowed us to further estimate when the transmission event would have taken place if either partner was the source, and how old both partners were at that time.

### Reconstruction of direction of transmission

The likely direction of transmission for each pair was assessed using two independent methods, firstly the phylogenetic topology,^11^ and secondly comparison of estimated infection dates (see appendix p 2). Where both methods assigned the same direction of transmission, or one assigned a direction and the other returned an indeterminate result, we took the pair forwards as one with a determined direction of transmission. We excluded pairs where the recipient’s estimated time of infection was outside the timeframe of the PopART trial (November, 2013 to December, 2018).

### Classification of drug resistance mutations

Drug resistance to first-line adult ART was predicted based on detection of mutations from the Stanford HIV Drug Resistance Database scoring system (HIVdb version 8.9.1)^20^ with the following cutoffs: 0–14: wild type or susceptible; 15–29: low-level resistance; 30 and above: high-level resistance. See the appendix (p 2) for further details.

### Characteristics of sources and demographic weighting

For each directed pair, we characterised the source partner by sex, age group (in 5-year bands), viral drug resistance mutations, and whether they were registered in a different community from their recipient. Sampling bias is an important consideration in a source attribution study. We observed that the study could be viewed as a survey in which recipients of infection provided information about their sources. Thus, methods for adjusting for unrepresentative sampling from survey methodology are appropriate, and we used iterative proportional fitting (“raking”) as implemented in the R *anesrake* package to weight the population of recipients to be demographically representative of the HIV-positive population of individuals in the trial, in regard to four variables: sex, birth cohort (pre-1960, post-1994, and seven bins of 5 years covering the intervening period), community, and marital status. The target population distribution of the former three variables was estimated from the PopART-IBM individual-based model^21^ while the last was calculated from the marital status of HIV-infected individuals in the 2018 Zambia Demographic and Health Survey.^22^ Proportions of sources with a particular characteristic (eg, age group) were then calculated by using the sum of their recipients’ weights as numerator, instead of the simple number of pairs. 95% CIs for weighted proportions were calculated using the R *survey* package.

### Sensitivity analyses

Three sensitivity analyses were performed. In the first, we used only samples for which we had a consensus call on 75% of the HIV genome, rather than 50%. In the second, we analysed samples from the health-care facilities only. In the third, we reconstructed the direction of transmission using the phylogenetic topology only.

### Role of the funding source

Representatives from the US National Institutes of Health (NIH) attended meetings concerned with study design and data analysis and interpretation. They had no role in the writing of this report. No other funders of the study had any role in study design, data collection, data analysis, data interpretation, writing of the report, or the decision to submit for publication.

## Results

**Figure 1:**
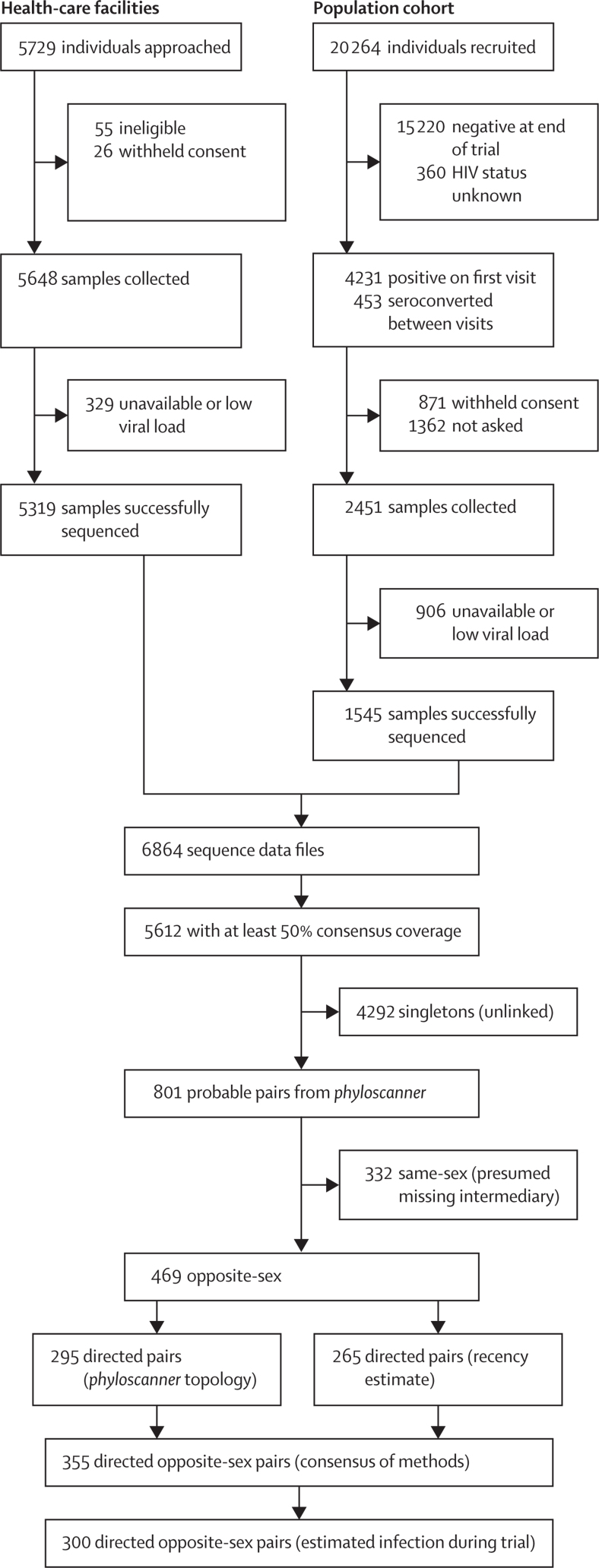
Flowchart depicting determination of transmission pairs from recruited participants from the nine Zambian communities

**Figure 2:**
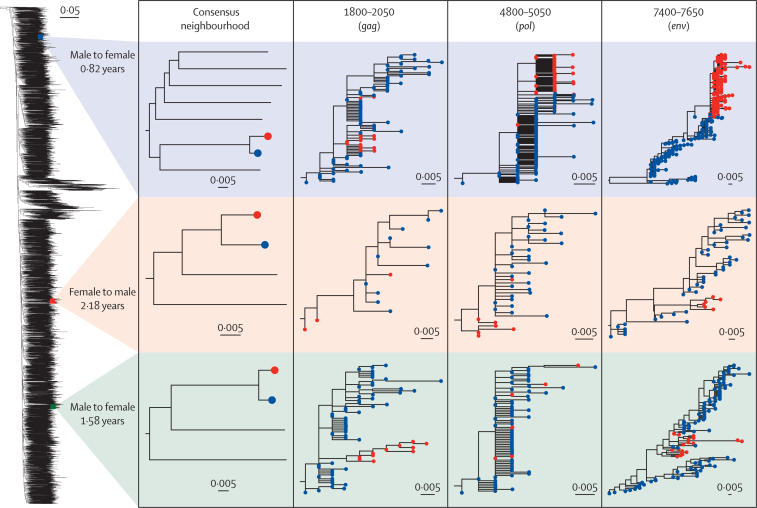
Example transmission pairs and windows From the consensus phylogeny of 5612 full-genome sequences, three transmission pairs are enlarged. Tips for the male individual are in blue and the female in red. Annotations indicate the reconstructed direction of transmission and the estimated time from infection to sampling of the recipient. The first column displays the position of each pair among its immediate neighbours in the consensus tree. The remaining three columns are example *phyloscanner* windows (coordinates and genes are given) and the within-host phylogenies for the pair in each. Note that the topological direction of transmission is identified by summarising patterns over all windows and thus some individual trees will not match it (eg, row 2, column 4). Tree branches are in units of substitutions per site (see scale bars for each tree).

Sampling yielded 6864 genomic sequences. We analysed 5612 for which 50% coverage of the consensus genome was obtained (figure 1, table, appendix p 6). 5263 of these (93·8%) were assigned subtype C by REGA, with 17 subtype A, two subtype G, and the rest either circulating recombinant forms or unique recombinants. The *phyloscanner* procedure identified a total of 801 pairs, of which 469 were opposite-sex, 242 female-to-female, and 90 male-to-male. The same-sex pairs are likely to be mostly or entirely erroneously linked (see Discussion), and as our focus was anyway on the heterosexual epidemic, we analysed the opposite-sex set only. See figure 2 for the consensus phylogeny and examples of within-host phylogenies in a selection of windows for three pairs. Histograms of the estimated times from infection to sampling of all included participants and those identified as sources of transmission in the analysis can be found in the appendix (p 5).

**Table:** Demographic and other characteristics of all eligible participants, and probable sources and recipients in the reconstructed transmission pairs

	All eligible participants (n=5612)	Sources (n=284)	Recipients (n=300)
**Trial arm**			
A	1802 (32·1%)	93 (32·7%)	99 (33·0%)
B	1758 (31·3%)	80 (28·2%)	86 (28·7%)
C	2051 (36·6%)	111 (39·1%)	115 (38·3%)
**Cohort**			
Health-care facilities	4685 (83·5%)	258 (90·8%)	274 (91·3%)
Population cohort	926 (16·5%)	26 (9·2%)	26 (8·7%)
**Sex**			
Female	3334 (59·4%)	121 (42·6%)	170 (56·7%)
Male	2277 (40·6%)	163 (57·4%)	130 (43·3%)
**Year of sampling**			
2013	1 (<0·1%)	0	0
2014	419 (7·5%)	16 (5·6%)	1 (0·3%)
2015	144 (2·6%)	3 (1·1%)	5 (1·7%)
2016	1703 (30·4%)	84 (29·6%)	92 (30·7%)
2017	2483 (44·3%)	130 (45·8%)	143 (47·7%)
2018	861 (15·3%)	51 (18·0%)	59 (19·7%)
**Age at sampling, years**			
10–19	202 (3·6%)	7 (2·5%)	12 (4·0%)
20–29	2087 (37·2%)	110 (38·7%)	154 (51·3%)
30–39	2109 (37·6%)	119 (41·9%)	89 (29·7%)
40–49	889 (15·8%)	37 (13·0%)	33 (11·0%)
50–59	244 (4·3%)	8 (2·8%)	10 (3·3%)
60–69	61 (1·1%)	3 (1·1%)	1 (0·3%)
≥70	12 (0·2%)	0	1 (0·3%)
Unknown	7 (0·1%)	0	0
**Marital status at sampling**			
Divorced or separated	1104 (19·7%)	37 (13%)	44 (14·7%)
Married or living as married	3086 (55%)	209 (73·6%)	204 (68·0%)
Never married	973 (17·3%)	33 (11·6%)	40 (13·3%)
Widowed	417 (7·4%)	3 (1·1%)	12 (4·0%)
Unknown	31 (0·6%)	2 (0·7%)	0

14 sources had multiple probable recipients.

The phylogenetic topology assigned a direction of transmission to 295 pairs, while 265 were called by comparison of the time of infection estimates. 145 were called by both methods, with 25 excluded due to conflicting results. When combined, the analysis yielded 355 probable directed transmission pairs. For 300, transmission was estimated to have occurred during the trial period (figure 1). This was consistent with a power calculation conducted before the study to determine the number of transmission pairs required to characterise sources of infection (see appendix p 2). 284 (94·7%) of the 300 pairs were subtype C for both partners. The remainder were called as recombinants in one or both cases, but C was always one of the parents (see appendix p 13).

The table outlines the characteristics of the individuals involved in the pairs; an extended version is in the appendix (pp 7–9). Note that there are fewer probable sources identified than recipients, as 14 individuals were reconstructed as infecting multiple others. 13 of these (seven males and six females) were probable sources for two other individuals, and one (female) for four others. We emphasise that the principal unit of our analysis is the transmission, and that characteristics such as ages at time of transmission and the source residing in a different community to the recipient will not necessarily be consistent between two transmissions even with the same source. We are exploring the characteristics of the sources involved in 300 distinct transmissions, rather than those of 300 unique sources.

**Figure 3:**
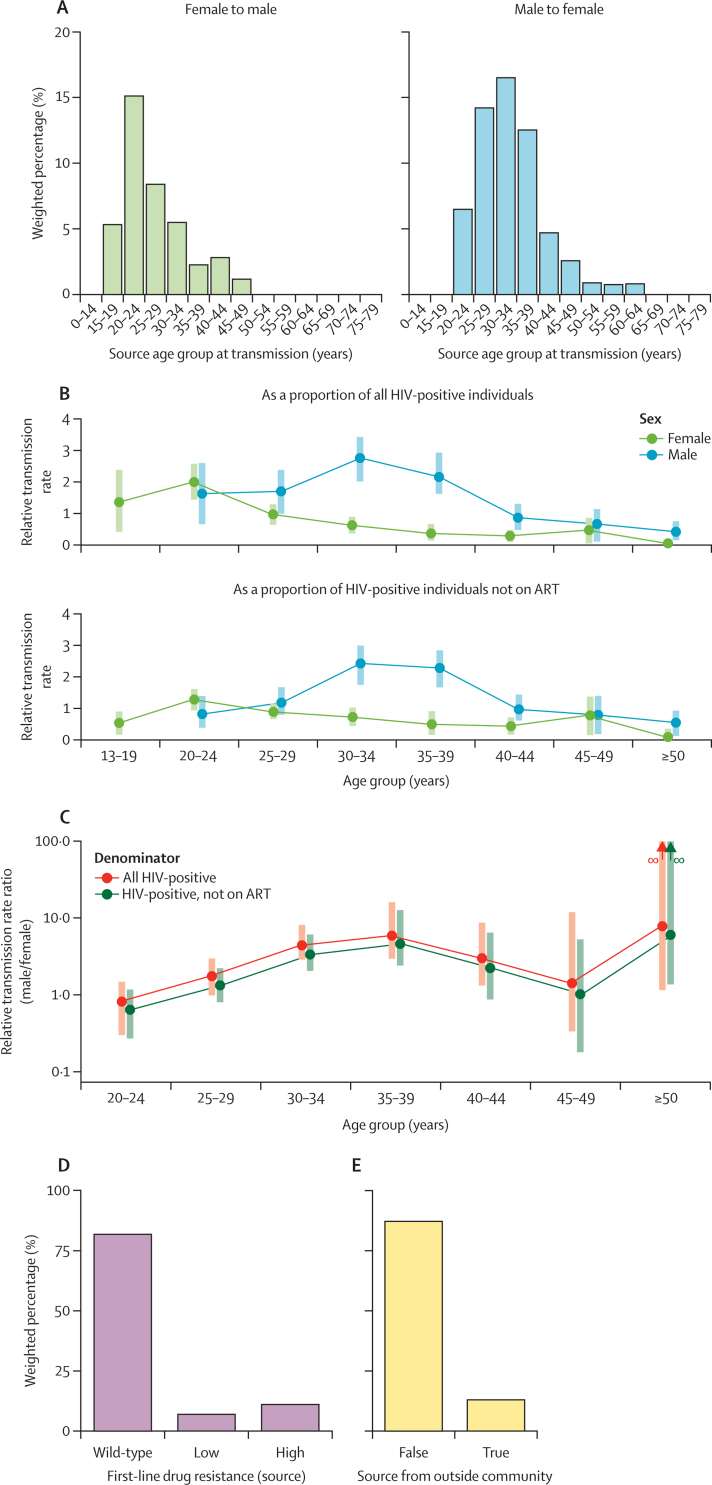
Characteristics of sources of transmission (A) Age profile (at the estimated time of transmission) of the sources of infection for participants estimated to have been infected during the trial period. Counts were weighted to make the recipient population representative of the HIV-positive population during the trial. (B) For different age and sex demographics, the ratio of the proportion of sources in that group to the proportion in the same group of (top) all HIV-positive individuals or (bottom) all HIV-positive individuals not on ART. Ages are calculated as of July 1, 2017. Shaded bars are 95% CIs determined by non-parametric bootstrap. We identified no male sources in the 13–19 years age group and thus this estimate is omitted. (C) Ratios for the relative contributions of male and female sources by age group (as calculated for panel B). Shaded bars are 95% CIs as before. The bootstrapped confidence intervals for the ≥50 years age group extend upwards to infinity. (D) First-line drug resistance profiles of sources. (E) Proportions of infections from sources residing in a different community from the recipient. ART=antiretroviral therapy.

Pre-weighting, 170 (56·7%) transmissions were from male to female participants; weighting brought this to 178 (59·4%). The age distributions of the individuals identified as probable sources, both male and female, showed the highest rates of transmission coming from individuals between 20 and 39 years of age: 130 (95% CI 114–144; 72·9%) of the 178 male sources were between 25 and 39 years of age at the estimated time of transmission, representing 43·3% of the 300 transmissions, while 87 (75–98; 71·6%) of 122 female sources were between 20 and 34 years of age, representing 29·0% (figure 3A). Unless otherwise stated, figures given in this section are all weighted to adjust for sampling bias as described in the Methods. Weighted counts are rounded to the nearest whole number. In the PopART-IBM simulation, 18 094 (19·4%) of 93 272 infected individuals in 2017 were men aged 25–39 years, while 25 443 (27·3%) were women aged 20–34 years. The median age at time of transmission was 32 years (range 20–64) for male sources and 25 years (17–49) for female sources (figure 3A). There was no evidence of a difference in the ages of sources (weighted Kruskal-Wallis test p=0·98 for male-to-female pairs, p=0·48 for female-to-male pairs) between trial arms.

We calculated relative transmission rates among demographic groups, defined as the ratio of the proportion of sources identified from a group to that group’s proportion of the overall HIV-positive population, as estimated by PopART-IBM (see appendix pp 2–3). For men, this was 1·44 (bootstrapped 95% CI 1·43–1·5) while for women it was 0·69 (0·65–0·7). The ratio of these two numbers is 2·09 (2·06–2·29), indicating that HIV-positive males were contributing more than twice as many new infections as HIV-positive females, per person. The ratio was 1·79 (1·78–1·95) when the group’s proportion of the HIV-positive population not on ART, instead of the total HIV-positive population, was used as a denominator, indicating that even when adjusting for ART coverage, men infected more women per person than vice versa.

Figures 3B and 3C show these statistics for both denominators stratified by age group. The peak relative rate among females was observed in the 20–24 years age group (2·0 compared with the HIV-positive population, 95% CI 1·41–2·67) while for males it was the 30–34 years age group (2·76; 2·09–3·37). The male-to-female ratio was 4·38 (2·8–8·25) in the 30–34 years age group and 5·87 (2·78–15·8) in the 35–39 years age group. This ratio was above 1 in all age groups other than 20–24 years but the confidence interval included 1 for the 20–24, 25–29, and 45–49 years age groups for both denominators.

A weighted total of 12 (3·8%) sources had an unknown drug resistance profile (due to poor genome coverage at relevant sites). Of the remaining 288, 236 (95% CI 220–250; 81·9%) carried drug-sensitive viruses, while 20 (12–32; 7·0%) showed low-level resistance to first-line ART and 32 (21–46; 11·1%) high-level resistance (figure 3D). Thus some measure of resistance was seen in 52 (38–69; 18·1%) sources. Again, there was no evidence of a difference in the distribution of mutations with respect to the trial arm of the recipient (χ^2^ test p=0·32). For comparison, of 285 recipients with non-missing data, 227 (209–242; 79·7%), 31 (21–46; 11·0%), and 27 (16–40; 9·3%) recipients had virus that was respectively sensitive, low-level, or high-level resistant, in line with a previously published study on resistance.^23^

Lastly, we determined the fraction of transmission pairs for which the source lived in a different community from the recipient (figure 3E). A weighted total of 260 of 300 (95% CI 243–274; 86·8%) transmissions occurred within the same community, compared with 40 (26–57; 13·2%) that did not. Contrary to the other variables, here there was some evidence of a difference in the proportion of transmissions coming from outside the community by trial arm (χ^2^ test p=0·035), with a rounded total of 10 of 91 (11·2%) from outside the community in arm A, 20 of 100 (20·1%) in arm B, and 9 of 109 (8·5%) in arm C.

**Figure 4:**
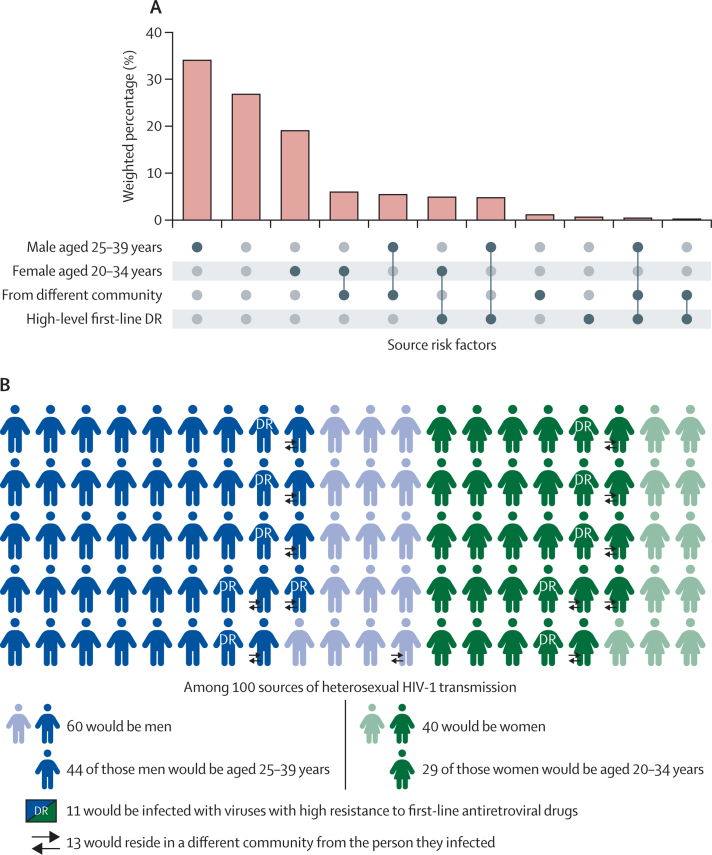
Combined characteristics of source individuals (A) Distribution of all combinations of four key risk factors among the set of directed opposite-sex pairs determined to have been infected during the trial period. Each bar represents a group of sources whose characteristics are defined by the combination of black dots on the x-axis. Bars are weighted as in previous plots. (B) Pictorial representation of the expected characteristics of 100 sources of transmission. Note that as all transmission is assumed to be heterosexual, the 60 men and 40 women will have infected, respectively, 60 women and 40 men. DR=drug resistance.

We next sought to understand the extent to which characteristics of sources might occur disproportionately often in combinations. To do this, we ranked each combination according to the fraction of transmissions in which they played a role (figure 4A). The highest fraction originated from males aged 25–39 years who lived in the same community as their recipient and had no viral resistance to first-line ART. They were the sources for 1·78 times as many transmissions as the female risk group aged 20–34 years with the same characteristics and 1·18 times as many transmissions as all sources outside these two age groups. There was no group that disproportionately combined several of the risk factors, as real values were generally very close to values expected if risk factors present in the population of sources had been randomly assigned to this population (appendix p 4).

Results of the three sensitivity analyses, which do not differ markedly from those of the main analysis, can be found in the appendix (pp 4, 9).

## Discussion

We identified characteristics of likely sources of HIV-1 transmission in Zambia between 2014 and 2019. The aim of the study is to provide policy makers with data on where transmission is still occurring in a generalised epidemic in sub-Saharan Africa, despite delivery of a UTT intervention, and advise where prevention efforts should be focused.

We identified men aged 25–39 years as the group most commonly transmitting HIV-1. They were the source of 43·3% of transmissions (figure 4B), despite males aged 25–39 years representing only 19·4% of prevalent cases in 2017. 29% of transmissions were attributable to women aged 20–34 years. Age distributions of sources were wide, suggesting that transmitters were not confined to any small demographic category. 2·09 times as many infections were attributable to male sources as to females, and when stratified by age this number rose to 4·38 for men and women in the 30–34 years age group and to 5·87 in the 35–39 years age group. Men in these age groups were notably less likely than other demographic groups to be linked to care and on ART.^24^ A similar phylogenetics study conducted as part of the Ya Tsie trial in Botswana largely concurred with this finding, although estimated ages for sources were somewhat greater.^25^ That study, however, recorded ages at time of sampling, with no attempt to estimate time of infection. These results highlight the need for more intensive efforts in UTT programmes to achieve high and sustained ART coverage and testing for men aged 25–39 years; a programme that leaves a deficit in coverage for the demographic group responsible for the greatest proportion of transmissions might be viewed as insufficiently universal. It also suggests that pre-exposure prophylaxis services should also be considered for the partners of these men.^26^

Much prevention effort on HIV in sub-Saharan Africa has focused on intergenerational relationships, older men, and the “sugar daddy” phenomenon.^27^ A previous phylogenetic study further suggested that the epidemic is maintained through a renewal process, in which young women are infected by considerably older men, who had been infected by women of a similar age to themselves.^28^ Policies aimed at breaking this cycle have been promoted by UNAIDS and PEPFAR, in particular with the DREAMS programme. We find little evidence for this cycle or for intergenerational relationships being major drivers of transmission, with the median age of female sources being 25 years and that of males 32 years. While we do find that a significant minority of transmissions came from outside the community, this is nevertheless a minority, casting doubt, at least in this setting, on another commonly hypothesised driver of new transmissions, human mobility.^29^ Our results in totality indicate that infections are predominantly local and disproportionately from men.

11·1% and 7·0% of sources’ viral populations were dominated by variants with high-level and low-level resistance against first-line ART, respectively. A caveat is that the precise timing of ART initiation was not known, and therefore some resistance could have been acquired in sources following transmission to the recipient. Nevertheless, this level of drug resistance mirrors the high percentage of first-line ART resistance reported by other studies in the past few years.^30^ Resistance against current first-line ART, including dolutegravir (which was not rolled out in Zambia at the time of this study), needs to be monitored by local surveillance teams as the availability of HIV drug resistance testing and next-generation sequencing in sub-Saharan Africa increases.

While a large majority of transmissions occurred between partners from the same community, 13·2% did not (figure 4B). This analysis cannot help but exclude pairs for which the source resided outside the trial population and thus could never be sampled, and so this estimate will be lower than the overall proportion of imports (and potentially much lower). Although movement does not necessarily compromise the effect of treatment as prevention, it does affect the ability of trials such as HPTN 071 to accurately measure its effect. Thus, as the primary analysis of the trial^2^ took no account of between-community transmission, even this probably underestimated figure indicates that HIV-1 incidence would have been further reduced if the PopART prevention package had been implemented country-wide. The complex statistical corrections required to assess the impact of the intervention taking into account between-community transmission are outside the scope of this study, but further genomic analysis is likely to lead to a higher estimate of its effectiveness.

A limitation of the *phyloscanner* procedure is that it is not able to rule out missing intermediaries in transmission, but for heterosexual epidemics this is easy to detect as it would result in observed same-sex pairs. Our 242 female-to-female pairs are exceptionally unlikely to represent genuine sexual transmission, but the 90 male-to-male examples could potentially be men who have sex with men (MSM). However, of all combinations of females in the dataset, a proportion of 5·2×10^–5^ (95% binomial CI 4·6×10^–5^–5·87×10^–5^) were linked by the analysis, compared with 4·09×10^–5^ (3·3×10^–5^–5·0×10^–5^) of males. As the female-to-female links must be erroneous, the overlapping confidence intervals suggest no evidence for an excess of male-to-male pairs that would imply a substantial contribution of cryptic MSM transmission to infections in the sampled population. We might be underestimating the amount of MSM transmission due to simple undersampling of MSM, especially if they were less likely to participate in the trial for reasons of stigma. Nevertheless, our results suggest that MSM are not widely integrated into heterosexual networks. The restriction to same-sex pairs also naturally removes any pairs with a single missing intermediary, increasing the specificity of *phyloscanner* as only situations of two or more missing intermediaries cannot be ruled out.

This study has limitations. This work was conducted in the context of the HPTN 071 trial, which potentially limits the generalisability of results to other settings. However, the trial did have a control arm, and the only variable that we explored whose distribution showed a clear difference between arms was transmission from outside the community, which was higher in intervention arms. This is consistent with the intervention reducing transmission within-community, resulting in a larger fraction of infections being caused by introductions. Nevertheless, we acknowledge that the conditions of even the control communities might have differed from those in other locations where no trial was taking place, for example because the presence of trial staff in the community might have some effect on health-care seeking behaviour in the population.

While between 13·0% and 35·2% of people living with HIV in the study communities were sampled, a proportion comparable to similar large studies in sub-Saharan Africa,^25^ and much larger than was possible in the quite recent past, the sample was still not fully representative of the whole HIV-positive population. In particular, the design restricted sampled individuals to one person per household in the population cohort, limiting our ability to identify within-household transmission pairs. This is mitigated by the collection of samples in the health-care facilities, which had no such restrictions, but might have introduced its own bias towards identifying individuals who presented to these facilities of their own volition. Our inclusion criterion for samples was simply that sequencing was successful above a quality threshold, which might also introduce some bias. In particular, the laboratory approach only yielded sequences for participants with more than 5000 viral copies per mL. This will exclude patients with lower viral loads, but still corresponds to 92·8% of participants with viral loads above 1500 copies per mL.^31^ Individuals on effective ART at the time of sampling would not usually be included, due to their specific exclusion in the health-care facilities and their low viral loads preventing sequence acquisition in the population cohort. We estimate that the contribution of MSM to transmission among the sampled members of this community is at most small, and hence that by including only opposite-sex pairs we analysed the transmission route responsible for sustaining infections in this population. Nevertheless, it remains possible that genuine examples of MSM transmission were detected, and we did not explore the characteristics of those pairs as it was impossible to distinguish them from heterosexual transmissions with a missing intermediary. A proper investigation of any MSM transmission in this or similar populations would require a different study design.

HPTN 071-02 (PopART) Phylogenetics is the largest HIV phylogenetic study conducted to date, the first large transmission study to be based on an a priori power calculation, and the most comprehensive study of characteristics of sources of HIV-1 infection in sub-Saharan Africa. The study highlights that residual transmission was not limited to coming from small risk groups. However, men aged 25–39 years were, as a group, responsible for a large share of transmissions and should be prioritised in prevention efforts, even if linking them to care requires more effort.

## Data sharing

Consensus sequences with PANGEA-specific identifiers, sex of participant, and year and country of sampling will be made available on Mendeley Data upon publication and on GenBank as soon as possible. A data dictionary for these fields, the consent form, and the analysis code will also be made available on Mendeley Data upon publication. Additional data fields and next-generation sequencing data can be requested via the data sharing procedures of the PANGEA consortium (see www.pangea-hiv.org). The requests are assessed by the PANGEA Steering Committee and data will usually be made available for non-commercial purposes unless there is a risk of participants and communities being harmed or stigmatised, or participants’ privacy being compromised. If multiple applicants are aiming to pursue the same analyses, they will be encouraged to coordinate efforts.

## Declaration of interests

DJD, SHE, CF, EP-M, and OR report grant funding from NIH for this work. MSC reports other NIH grant funding. SHE reports NIH funding for meetings and travel. CF and OR report grant funding from the Bill & Melinda Gates Foundation for this work. HA reports honoraria from the Global Fund. MSC reports payments from Medscape and UpToDate for written material. WP reports consulting fees from WHO. All other authors declare no competing interests.
