# Supplementary material for: Demographics of sources of HIV-1 transmission in Zambia: a molecular epidemiology analysis in the HPTN 071 PopART study
Source: Lancet Microbe. 2024 Jan;5(1):e62–71. doi: 10.1016/S2666-5247(23)00220-3 (PMC10789608; doi:10.1016/S2666-5247(23)00220-3)
Supplement: Supplementary appendix [file mmc1.pdf]

Appendix

Table of contents

1. Further sequencing details
2. Consensus phylogeny
3. Phyloscanner procedure
4. Reconstruction of direction of transmission by estimated time of infection
5. Power calculation for transmission pairs
6. Detection and classification of drug resistance mutations (further details)
7. Calculation of relative transmission rates
8. Figure S1: Extended version of figure 4A
9. Figure S2: Relative transmission rates calculated from the sensitivity analyses
10. Figure S3: Histograms of estimated times from infection to sampling
11. Table S1: Extended participants table
12. Table S2: Extended baseline demographics table
13. Table S3: Sensitivity analysis results
14. Table S4: Command line options for HIV-TRACE, IQ-TREE, and *phyloscanner*
15. Table S5: REGA subtyping results for individuals in transmission pairs
16. References

Further sequencing details

Plasma samples used for this study were derived from both residual EDTA treated blood collected for CD4 quantification, centrifuged at the local health care facility and then transported frozen to a central lab in Lusaka then shipped to Oxford for extraction and onward processing. Full details of the sequencing methods and bioinformatics have previously been published, along with a validation against a clinically accredited HIV drug resistance assay and HIV viral load assay[1], which demonstrated robust whole genome characterisations for samples with viral loads > 5000 RNA copies per ml. Water controls and negative plasma controls are included in each batch of 96 samples to monitor for physical contamination, along with a standard curve (a ten-fold serial dilution of a plasma-diluted culture stock of HXB2) for quantitative calibrations and additional monitoring for cross-contamination. Unique dual indexing allowed us to monitor for, and exclude, reads with unexpected index pairs that can arise because of index hopping or overly-dense optical clustering on the flow-cell.

Total RNA was extracted with magnetized silica from HIV-infected plasma lysed with guanidine thiocyanate and with ethanol washes and elution steps performed using the NUCLISENS easyMAG system (bioMérieux). The total 30 µl elution volume was reduced with Agencourt RNAClean XP (Beckman Coulter). Libraries retaining directionality were prepared using the SMARTer Stranded Total RNA-Seq kit v2 - Pico Input Mammalian (Clontech, TaKaRa Bio). Dual-indexed amplified cDNA libraries were carried out using in-house sets of 96 i7 and 96 i5 indexed primers. Details of the primers can be found elsewhere[2]. Equal volumes of each amplified library were pooled in 96-plex. A total of 500 ng of pooled libraries was hybridized (SeqCap EZ reagent kit, Roche) to a mixture of custom HIV-specific biotinylated 120-mer oligonucleotides (xGen Lockdown Probes, Integrated DNA Technologies), then pulled down with streptavidin-conjugated beads. Unbound DNA was washed off the beads (SeqCap EZ hybridization and wash kit, Roche), and the captured libraries were PCR amplified to produce the final pool for sequencing using a MiSeq (Illumina) instrument with v3 chemistry for a read length up to 300 nt paired-end. Alternatively, up to 384 samples were sequenced on HiSeq 2500 set to Rapid run mode using HiSeq Rapid SBS kit v2 with maximum read lengths of 250 nt.

Consensus phylogeny

The consensus sequences output by *shiver* were each pairwise aligned to the subtype C reference genome used in *phyloscanner* (NCBI accession number AF443088.1) to generate a consensus alignment. A simian immunodeficiency virus sequence (NC_004455.1) was included to represent an outgroup. The consensus phylogeny in figure 2 was generated for this alignment using IQ-TREE 1.6.12[3] and the FreeRate nucleotide substitution model with four rate categories.

Phyloscanner procedure

We used a set of 898 genomic windows, each of 250 base pairs in length, spaced at regular intervals to span the entire HIV-1 genome. Alignment of the reads intersecting each window, together with a set of 22 reference sequences, was performed with *phyloscanner*, and phylogenetic reconstruction performed on each alignment using IQ-TREE 1.6.12[4] and the FreeRate nucleotide substitution model with six rate categories. For a full list of command-line options used in HIV-TRACE, *phyloscanner* and IQ-TREE, see Table S4. Blacklisting, with a *k* parameter of 15, was performed to eliminate likely contaminants reads from the phylogenies. The 22 reference sequences were the standard references from *phyloscanner* for HIV-1 types B and C, together with a random sample of 20 PopART consensus sequences.

Likely transmission pairs were identified from the 898 phylogenies where their subtrees lay within a normalised patristic distance threshold of 0·02 substitutions per site in at least 50% of windows, after adjusting for windows with missing coverage as in[5]. Topological determination of the direction of transmission also used *phyloscanner*. Directionality was called if it was established in at least 33% of windows (adjusted for missing windows as before).

In a small number of cases where participants were reconstructed as the probable recipient of more than one transmission, the source was taken as the one with the greatest number of *phyloscanner* windows with normalised patristic distance less than 0·02.

Reconstruction of direction of transmission by estimated time of infection

Phylo-TSI gives both a point estimate for the date of infection and a standard deviation of the estimate; these were used to fit normal distributions. Directionality was called if the distributions for the two infection date estimates had an overlap of less than 20% area under the curve.

Power calculation for transmission pairs

The power calculation outlined in the PopART Phylogenetics study protocol (p55-57 of [6]) predicted that a total of 269 transmission pairs from incident cases would be identifiable, under the assumption that of all the transmission pairs for which both individuals provided sequences, 75% could be identified as pairs by the analysis. In the event, we identified 300 such pairs. This is despite the dataset consisting of 6,865 sequences, considerably lower than the protocol's prediction of the acquisition of 9,156 (p31).

Detection and classification of drug resistance mutations (further details)

A bioinformatic pipeline, drmSEQ, was used to predict drug resistance to first-line adult ART based on detection of mutations in the Illumina reads generated by veSEQ-HIV using the Stanford HIV Drug Resistance Database scoring system (HIVdb version 8.9.1)[7] as follows: wild type/susceptible for scores 0-14 -, low-level resistance for scores 15-29, and high-level resistance for scores 30 and above. The method has been previously validated against an FDA-approved drug resistance assay[1]. First-line adult ART national guidance at the time of sampling was non-nucleoside inhibitor efavirenz in combination with nucleoside inhibitors including abacavir, AZT, D4T, DDI, FTC, 3TC or Tenofovir.

Mutations were reported when detected in three or more PCR-deduplicated reads and 5% or more reads spanning each site. Drug Resistance was reported as 'unknown' when fewer than 50% of sites relevant to each drug reached the minimum coverage threshold of 3 or more PCR-deduplicated reads.

Calculation of relative transmission rates

The relative contribution of demographic groups to transmission, compared to their share of the overall population, was determined as follows. Suppose a represents an age group and s a sex. (PopART-IBM age groups are in five-year increments except that there exists a 13-14 group. We combined this with the 15-19 category to give a 13-19 group, and also combined all age groups over 50 together into one category.) We calculated y_{sa}, the proportion of all HIV+ individuals in 2017 belonging to a and s according to the IBM, and k_{sa}, the proportion of our pairs, weighted as described above, where the source belonged to s and the recipient belonged to a in July 2017 (regardless of their estimated date of infection). We then calculated:

$$\rho_{sa} = \frac{k_{sa}}{y_{sa}}$$

This represents the relative rate of transmissions coming from the combination of a and s. It is above 1 if that demographic category is overrepresented amongst sources, and less than 1 if it is underrepresented.

We can also calculate ρ_{sa} across all age groups to get the statistics ρ_F for all females and ρ_M for all males. Then

$$\frac{\rho_M}{\rho_F} = \frac{k_M}{y_M} / \frac{k_F}{y_F}$$

represents the ratio of the number of male sources per infected male to the number of female sources per infected female. This ratio can also be calculated individually in each age group.

For y_{sa} we used the IBM-derived HIV+ population. As the vast majority of new infections will come from people not currently on ART, an alternative statistic is $\tilde{y}_{sa}$, representing the proportion of HIV positive individuals not currently on ART who are in both a and s. The relative rate using $\tilde{y}_{sa}$ as a denominator, $\tilde{\rho}_{sa}$, is calculated in the same way.

To derive confidence intervals for these statistics, we performed a non-parametric bootstrap by sampling the set of observed pairs 200 times with replacement, re-weighting this new dataset by iterative proportional fitting, and calculating k_{sa} again. The limits of the confidence intervals were recorded as the 0.025 and 0.975 quantiles of the values of each statistic.

Figure S1. Extended version of figure 4. Actual distribution of risk factors compared to the distribution expected if they had been allocated randomly to sources based on risk factor percentages identified in the source population.

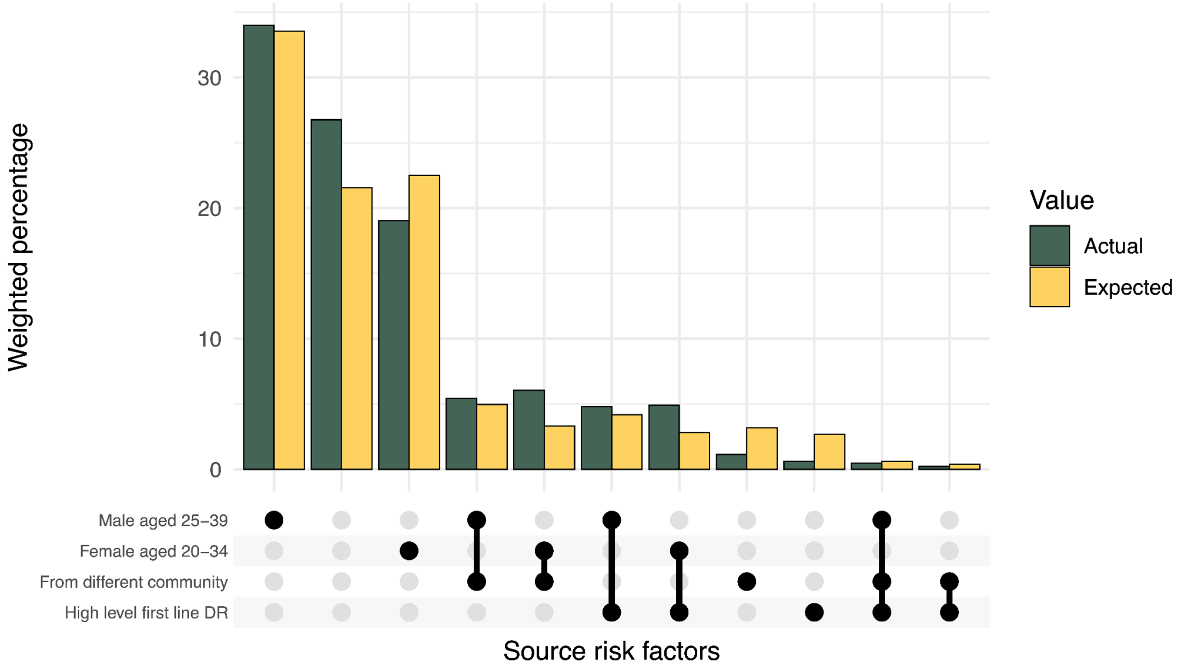

Figure S2: Relative transmission rates calculated from the sensitivity analyses. In the first two rows, the the ratio of the proportion of sources in an age group that are female (top) or male (bottom) to the proportion in the same group of (left) all HIV+ individuals or (right) all HIV+ individuals not on ART. Ages are calculated as of July 2017. We identified no male sources in the 13-19 age group and thus this estimate is omitted. Line and point colours represent the main analysis and the three sensitivity analyses. The bottom graphs depict the Ratios of the relative contributions of male and female sources by age group.

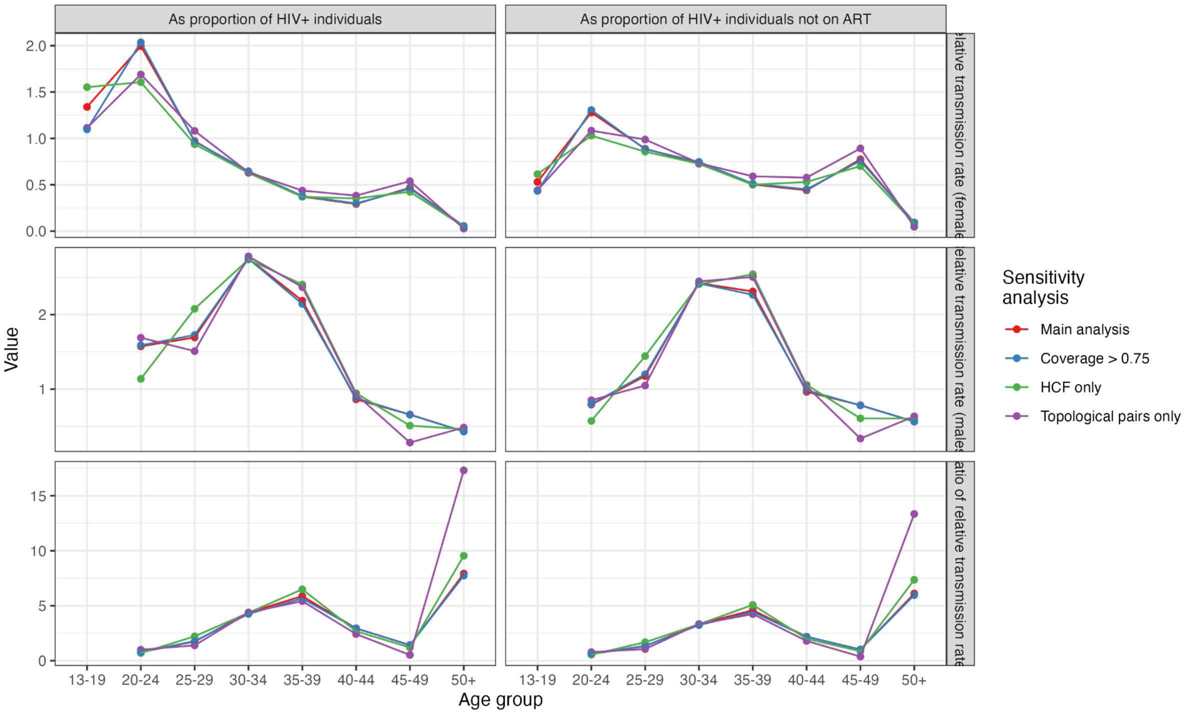

Figure S3: Histograms of estimated times from infection to sampling. (A) All 5,612 included participants. B) The 300 recipients in the reconstructed transmission pairs.

A

B

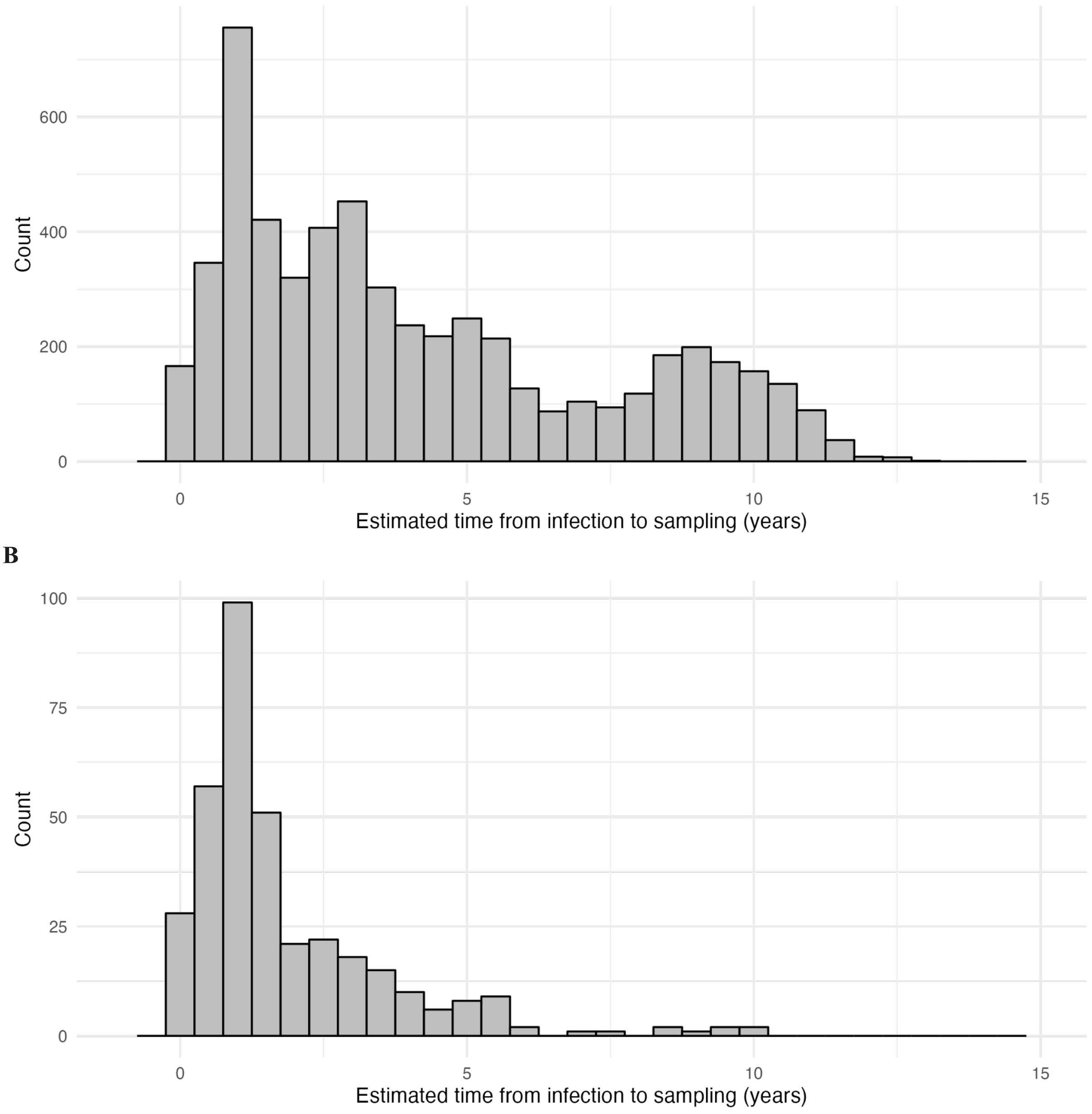

Table S1. Extended participants table. HIV-positive study participants, sequence availability, and inferred sources and recipients in probable transmission pairs, by arm of the study. HCF = health care facilities, PC seroconverters = participants in the population cohort who seroconverted during the trial period. PC0, PC12, PC24 = population cohort participants, HIV positive at baseline, recruited at the start of the trial (PC0) after 12 months (PC12N) and after 24 months (PC24N).

Recruitment	Total HIV positive participants	Participants successfully sequenced	Sequences meeting quality threshold	Sources	Recipients
HCF	5728	5319	4686	258	274
PC seroconverters	453	180	171	2	17
PC0	3065	843	512	18	2
PC12N	738	247	110	3	2
PC24N	428	275	133	3	5

Table S2. Demographic and other characteristics of all eligible participants, and sources and recipients in the reconstructed transmission pairs, by trial arm and overall. A total of 300 pairs were found, but 14 sources had multiple probable recipients.

Variable	Value	All eligible participants				Sources				Recipients			
		Arm			Total	Arm			Total	Arm			Total
		A	B	C		A	B	C		A	B	C	
Cohort	HCF	1474 (81·8%)	1492 (84·9%)	1719 (83·8%)	4685 (83·5%)	85 (91·4%)	68 (85·0%)	105 (94·6%)	258 (90·8%)	85 (85·9%)	83 (96·5%)	106 (92·2%)	274 (91·3%)
	PC	328 (18·2%)	266 (15·1%)	332 (16·2%)	926 (16·5%)	8 (8·6%)	12 (15·0%)	6 (5·4%)	26 (9·2%)	14 (14·1%)	3 (3·5%)	9 (7·8%)	26 (8·7%)
Sex	F	1100 (61·0%)	1033 (58·8%)	1201 (58·6%)	3334 (59·4%)	43 (46·2%)	38 (47·5%)	40 (36·0%)	121 (42·6%)	53 (53·5%)	47 (54·7%)	70 (60·9%)	170 (56·7%)
	M	702 (39·0%)	725 (41·2%)	850 (41·4%)	2277 (40·6%)	50 (53·8%)	42 (52·5%)	71 (64·0%)	163 (57·4%)	46 (46·5%)	39 (45·3%)	45 (39·1%)	130 (43·3%)
Year of birth	1935-1939	0 (0·0%)	1 (0·1%)	2 (0·1%)	3 (0·1%)	0 (0·0%)	0 (0·0%)	0 (0·0%)	0 (0·0%)	0 (0·0%)	0 (0·0%)	1 (0·9%)	1 (0·3%)
	1940-1944	1 (0·1%)	3 (0·2%)	1 (0·0%)	5 (0·1%)	0 (0·0%)	0 (0·0%)	0 (0·0%)	0 (0·0%)	0 (0·0%)	0 (0·0%)	0 (0·0%)	0 (0·0%)
	1945-1949	5 (0·3%)	3 (0·2%)	6 (0·3%)	14 (0·2%)	0 (0·0%)	0 (0·0%)	0 (0·0%)	0 (0·0%)	0 (0·0%)	0 (0·0%)	0 (0·0%)	0 (0·0%)
	1950-1954	13 (0·7%)	7 (0·4%)	8 (0·4%)	28 (0·5%)	1 (1·1%)	1 (1·2%)	0 (0·0%)	2 (0·7%)	0 (0·0%)	0 (0·0%)	0 (0·0%)	0 (0·0%)
	1955-1959	23 (1·3%)	28 (1·6%)	22 (1·1%)	73 (1·3%)	0 (0·0%)	1 (1·2%)	0 (0·0%)	1 (0·4%)	2 (2·0%)	2 (2·3%)	0 (0·0%)	4 (1·3%)
	1960-1964	34 (1·9%)	43 (2·4%)	45 (2·2%)	122 (2·2%)	1 (1·1%)	1 (1·2%)	1 (0·9%)	3 (1·1%)	1 (1·0%)	2 (2·3%)	0 (0·0%)	3 (1·0%)
	1965-1969	71 (3·9%)	70 (4·0%)	80 (3·9%)	221 (3·9%)	5 (5·4%)	1 (1·2%)	4 (3·6%)	10 (3·5%)	2 (2·0%)	5 (5·8%)	2 (1·7%)	9 (3·0%)
	1970-1974	137 (7·6%)	149 (8·5%)	175 (8·5%)	461 (8·2%)	10 (10·8%)	4 (5·0%)	6 (5·4%)	20 (7·0%)	3 (3·0%)	1 (1·2%)	3 (2·6%)	7 (2·3%)
	1975-1979	241 (13·4%)	242 (13·8%)	288 (14·0%)	771 (13·7%)	7 (7·5%)	8 (10·0%)	18 (16·2%)	33 (11·6%)	14 (14·1%)	11 (12·8%)	6 (5·2%)	31 (10·3%)
	1980-1984	359 (19·9%)	353 (20·1%)	405 (19·7%)	1117 (19·9%)	18 (19·4%)	23 (28·7%)	26 (23·4%)	67 (23·6%)	11 (11·1%)	14 (16·3%)	23 (20·0%)	48 (16·0%)
	1985-1989	370 (20·5%)	358 (20·4%)	437 (21·3%)	1165 (20·8%)	21 (22·6%)	15 (18·8%)	21 (18·9%)	57 (20·1%)	25 (25·3%)	15 (17·4%)	33 (28·7%)	73 (24·3%)

	1990-1994	365 (20·3%)	348 (19·8%)	391 (19·1%)	1104 (19·7%)	21 (22·6%)	21 (26·2%)	22 (19·8%)	64 (22·5%)	35 (35·4%)	26 (30·2%)	29 (25·2%)	90 (30·0%)
	1995-1999	180 (10·0%)	152 (8·6%)	186 (9·1%)	518 (9·2%)	9 (9·7%)	5 (6·2%)	13 (11·7%)	27 (9·5%)	6 (6·1%)	10 (11·6%)	18 (15·7%)	34 (11·3%)
	2000-2004	2 (0·1%)	0 (0·0%)	0 (0·0%)	2 (0·0%)	0 (0·0%)	0 (0·0%)	0 (0·0%)	0 (0·0%)	0 (0·0%)	0 (0·0%)	0 (0·0%)	0 (0·0%)
	Unknown	1 (0·1%)	1 (0·1%)	5 (0·2%)	7 (0·1%)	0 (0·0%)	0 (0·0%)	0 (0·0%)	0 (0·0%)	0 (0·0%)	0 (0·0%)	0 (0·0%)	0 (0·0%)
Year of sampling	2013	1 (0·1%)	0 (0·0%)	0 (0·0%)	1 (0·0%)	0 (0·0%)	0 (0·0%)	0 (0·0%)	0 (0·0%)	0 (0·0%)	0 (0·0%)	0 (0·0%)	0 (0·0%)
	2014	124 (6·9%)	148 (8·4%)	147 (7·2%)	419 (7·5%)	4 (4·3%)	9 (11·2%)	3 (2·7%)	16 (5·6%)	0 (0·0%)	0 (0·0%)	1 (0·9%)	1 (0·3%)
	2015	48 (2·7%)	44 (2·5%)	52 (2·5%)	144 (2·6%)	2 (2·2%)	0 (0·0%)	1 (0·9%)	3 (1·1%)	3 (3·0%)	0 (0·0%)	2 (1·7%)	5 (1·7%)
	2016	466 (25·9%)	651 (37·0%)	586 (28·6%)	1703 (30·4%)	21 (22·6%)	30 (37·5%)	33 (29·7%)	84 (29·6%)	29 (29·3%)	36 (41·9%)	27 (23·5%)	92 (30·7%)
	2017	850 (47·2%)	717 (40·8%)	916 (44·7%)	2483 (44·3%)	48 (51·6%)	34 (42·5%)	48 (43·2%)	130 (45·8%)	50 (50·5%)	40 (46·5%)	53 (46·1%)	143 (47·7%)
	2018	313 (17·4%)	198 (11·3%)	350 (17·1%)	861 (15·3%)	18 (19·4%)	7 (8·8%)	26 (23·4%)	51 (18·0%)	17 (17·2%)	10 (11·6%)	32 (27·8%)	59 (19·7%)
Marital status at sampling	Never married	314 (17·4%)	277 (15·8%)	382 (18·6%)	973 (17·3%)	12 (12·9%)	8 (10·0%)	13 (11·7%)	33 (11·6%)	10 (10·1%)	14 (16·3%)	16 (13·9%)	40 (13·3%)
	Married/living as married	970 (53·8%)	975 (55·5%)	1141 (55·6%)	3086 (55·0%)	66 (71·0%)	60 (75·0%)	83 (74·8%)	209 (73·6%)	68 (68·7%)	56 (65·1%)	80 (69·6%)	204 (68·0%)
	Divorced/separated	360 (20·0%)	373 (21·2%)	371 (18·1%)	1104 (19·7%)	14 (15·1%)	11 (13·8%)	12 (10·8%)	37 (13·0%)	16 (16·2%)	14 (16·3%)	14 (12·2%)	44 (14·7%)
	Widowed	147 (8·2%)	125 (7·1%)	145 (7·1%)	417 (7·4%)	1 (1·1%)	1 (1·2%)	1 (0·9%)	3 (1·1%)	5 (5·1%)	2 (2·3%)	5 (4·3%)	12 (4·0%)
	Unknown	11 (0·6%)	8 (0·5%)	12 (0·6%)	31 (0·6%)	0 (0·0%)	0 (0·0%)	2 (1·8%)	2 (0·7%)	0 (0·0%)	0 (0·0%)	0 (0·0%)	0 (0·0%)
Age at sampling	10-19	72 (4·0%)	55 (3·1%)	75 (3·7%)	202 (3·6%)	3 (3·2%)	2 (2·5%)	2 (1·8%)	7 (2·5%)	0 (0·0%)	6 (7·0%)	6 (5·2%)	12 (4·0%)
	20-29	678 (37·6%)	669 (38·1%)	740 (36·1%)	2,087 (37·2%)	39 (41·9%)	28 (35·0%)	43 (38·7%)	110 (38·7%)	56 (56·6%)	39 (45·3%)	59 (51·3%)	154 (51·3%)
	30-39	671 (37·2%)	649 (36·9%)	789 (38·5%)	2,109 (37·6%)	32 (34·4%)	41 (51·2%)	46 (41·4%)	119 (41·9%)	25 (25·3%)	25 (29·1%)	39 (33·9%)	89 (29·7%)
	40-49	278 (15·4%)	279 (15·9%)	332 (16·2%)	889 (15·8%)	14 (15·1%)	6 (7·5%)	17 (15·3%)	37 (13·0%)	14 (14·1%)	11 (12·8%)	8 (7·0%)	33 (11·0%)
	50-59	72 (4·0%)	87 (4·9%)	85 (4·1%)	244 (4·3%)	4 (4·3%)	1 (1·2%)	3 (2·7%)	8 (2·8%)	3 (3·0%)	5 (5·8%)	2 (1·7%)	10 (3·3%)
	60-69	28 (1·6%)	14 (0·8%)	19 (0·9%)	61 (1·1%)	1 (1·1%)	2 (2·5%)	0 (0·0%)	3 (1·1%)	1 (1·0%)	0 (0·0%)	0 (0·0%)	1 (0·3%)
	70+	2 (0·1%)	4 (0·2%)	6 (0·3%)	12 (0·2%)	0 (0·0%)	0 (0·0%)	0 (0·0%)	0 (0·0%)	0 (0·0%)	0 (0·0%)	1 (0·9%)	1 (0·3%)

	Unknown	1<br>(0·1%)	1<br>(0·1%)	5<br>(0·2%)	7<br>(0·1%)	0<br>(0·0%)	0<br>(0·0%)	0<br>(0·0%)	0<br>(0·0%)	0<br>(0·0%)	0<br>(0·0%)	0<br>(0·0%)	0<br>(0·0%)
Estimated elapsed years	0	362<br>(20·1%)	280<br>(15·9%)	344<br>(16·8%)	986<br>(17·6%)	11<br>(11·8%)	9<br>(11·2%)	16<br>(14·4%)	36<br>(12·7%)	54<br>(54·5%)	41<br>(47·7%)	56<br>(48·7%)	151<br>(50·3%)
from infection to sampling	1	289<br>(16·0%)	283<br>(16·1%)	309<br>(15·1%)	881<br>(15·7%)	12<br>(12·9%)	11<br>(13·8%)	15<br>(13·5%)	38<br>(13·4%)	32<br>(32·3%)	24<br>(27·9%)	39<br>(33·9%)	95<br>(31·7%)
	2	259<br>(14·4%)	261<br>(14·8%)	274<br>(13·4%)	794<br>(14·2%)	18<br>(19·4%)	12<br>(15·0%)	17<br>(15·3%)	47<br>(16·5%)	9<br>(9·1%)	16<br>(18·6%)	16<br>(13·9%)	41<br>(13·7%)
	3	200<br>(11·1%)	201<br>(11·4%)	219<br>(10·7%)	620<br>(11·0%)	17<br>(18·3%)	12<br>(15·0%)	16<br>(14·4%)	45<br>(15·8%)	4<br>(4·0%)	5<br>(5·8%)	3<br>(2·6%)	12<br>(4·0%)
	4	136<br>(7·5%)	128<br>(7·3%)	200<br>(9·8%)	464<br>(8·3%)	4<br>(4·3%)	7<br>(8·8%)	9<br>(8·1%)	20<br>(7·0%)	0<br>(0·0%)	0<br>(0·0%)	1<br>(0·9%)	1<br>(0·3%)
	5	127<br>(7·0%)	145<br>(8·2%)	153<br>(7·5%)	425<br>(7·6%)	5<br>(5·4%)	5<br>(6·2%)	8<br>(7·2%)	18<br>(6·3%)	0<br>(0·0%)	0<br>(0·0%)	0<br>(0·0%)	0<br>(0·0%)
	6	58<br>(3·2%)	67<br>(3·8%)	61<br>(3·0%)	186<br>(3·3%)	6<br>(6·5%)	6<br>(7·5%)	5<br>(4·5%)	17<br>(6·0%)	0<br>(0·0%)	0<br>(0·0%)	0<br>(0·0%)	0<br>(0·0%)
	7	65<br>(3·6%)	53<br>(3·0%)	81<br>(3·9%)	199<br>(3·5%)	6<br>(6·5%)	3<br>(3·8%)	4<br>(3·6%)	13<br>(4·6%)	0<br>(0·0%)	0<br>(0·0%)	0<br>(0·0%)	0<br>(0·0%)
	8	100<br>(5·5%)	116<br>(6·6%)	129<br>(6·3%)	345<br>(6·1%)	6<br>(6·5%)	4<br>(5·0%)	4<br>(3·6%)	14<br>(4·9%)	0<br>(0·0%)	0<br>(0·0%)	0<br>(0·0%)	0<br>(0·0%)
	9	107<br>(5·9%)	117<br>(6·7%)	134<br>(6·5%)	358<br>(6·4%)	5<br>(5·4%)	7<br>(8·8%)	5<br>(4·5%)	17<br>(6·0%)	0<br>(0·0%)	0<br>(0·0%)	0<br>(0·0%)	0<br>(0·0%)
	10	72<br>(4·0%)	82<br>(4·7%)	111<br>(5·4%)	265<br>(4·7%)	0<br>(0·0%)	4<br>(5·0%)	8<br>(7·2%)	12<br>(4·2%)	0<br>(0·0%)	0<br>(0·0%)	0<br>(0·0%)	0<br>(0·0%)
	11	23<br>(1·3%)	22<br>(1·3%)	32<br>(1·6%)	77<br>(1·4%)	3<br>(3·2%)	0<br>(0·0%)	4<br>(3·6%)	7<br>(2·5%)	0<br>(0·0%)	0<br>(0·0%)	0<br>(0·0%)	0<br>(0·0%)
	12	4<br>(0·2%)	3<br>(0·2%)	4<br>(0·2%)	11<br>(0·2%)	0<br>(0·0%)	0<br>(0·0%)	0<br>(0·0%)	0<br>(0·0%)	0<br>(0·0%)	0<br>(0·0%)	0<br>(0·0%)	0<br>(0·0%)

Table S3. Sensitivity analyses. Key quantities from the main analysis and from the three sensitivity analyses. All quantities apart from pair counts are demographically weighted.

Variable	Dataset			
	**Main analysis**	**75% coverage**	**HCF only**	**Topological ascertainment only**
**Number of pairs**				
Female to male	130	128	105	101
Male to female	170	168	143	140
Total	300	296	248	241
**Source characteristics (proportion and 95% confidence interval)**				
Male aged 25-39 (proportion of total population)	0·433 (0·367-0·501)	0·431 (0·364-0·5)	0·473 (0·396-0·552)	0·435 (0·361-0·511)
Male aged 25-39 (proportion of males)	0·729 (0·64-0·806)	0·725 (0·635-0·803)	0·765 (0·675-0·841)	0·731 (0·632-0·816)
Female aged 20-34 (proportion of total population)	0·29 (0·235-0·351)	0·295 (0·238-0·356)	0·267 (0·205-0·335)	0·288 (0·225-0·357)
Female aged 20-34 (proportion of females)	0·716 (0·617-0·802)	0·726 (0·628-0·811)	0·698 (0·576-0·803)	0·71 (0·599-0·805)
Source in different community	0·132 (0·0871-0·189)	0·134 (0·087-0·194)	0·116 (0·0672-0·184)	0·125 (0·0763-0·189)
Source with wild-type virus	0·819 (0·762-0·868)	0·816 (0·758-0·865)	0·814 (0·747-0·869)	0·826 (0·766-0·877)
Source with low-level DRMs	0·0698 (0·0399-0·112)	0·071 (0·0409-0·113)	0·0641 (0·0318-0·113)	0·076 (0·0433-0·122)
Source with high-level DRMs	0·111 (0·0729-0·16)	0·113 (0·0743-0·163)	0·122 (0·0777-0·18)	0·0976 (0·0598-0·148)
Recipient with wild-type virus	0·797 (0·736-0·849)	0·801 (0·741-0·853)	0·789 (0·717-0·849)	0·766 (0·695-0·827)
Recipient with low-level DRMs	0·11 (0·0713-0·16)	0·105 (0·0677-0·154)	0·12 (0·0741-0·179)	0·133 (0·0873-0·192)
Recipient with high-level DRMs	0·0932 (0·0576-0·141)	0·0938 (0·0578-0·142)	0·0918 (0·0515-0·148)	0·101 (0·0591-0·158)
**Source ages (weighted median and weighted IQR)**				
Age of male sources	32·2 (28·5-36·7)	31·9 (28·4-36·7)	32·9 (29-36·4)	32·3 (28·8-36·3)
Age of female sources	25 (21·8-30·8)	25 (21·8-31)	25·7 (21·4-33)	26 (22·2-33·1)

Table S4: Command line options for HIV-TRACE, IQ-TREE, and the two phyloscanner commands. For further details, see the relevant package manuals.

Option	Meaning	Value	Value meaning/notes
HIV-TRACE			
--ambiguities	Handling of ambiguous nucleotides	average	Average all possible resolutions
--minoverlap	Minimum proportional overlap of sequences for inclusion in distance calculations	0·5	
--threshold	Distance threshold	0·04	
--curate	Contaminant screening	remove	Remove sequences that cluster with the reference
phyloscanner_make_trees			
--pairwise-align-to	Pairwise align all reads to this reference	<HXB2 sequence file>	

--excision-coords	Coordinates in the reference to be excised	823, 824, 825, 892, 893, 894, 907, 908, 909, 1012, 1013, 1014, 1156, 1157, 1158, 1384, 1385, 1386, 1444, 1445, 1446, 1930, 1931, 1932, 1957, 1958, 1959, 2014, 2015, 2016, 2023, 2024, 2025, 2080, 2081, 2082, 2134, 2135, 2136, 2191, 2192, 2193, 2280, 2281, 2282, 2283, 2284, 2285, 2298, 2299, 2300, 2310, 2311, 2312, 2316, 2317, 2318, 2319, 2320, 2321, 2322, 2323, 2324, 2340, 2341, 2342, 2346, 2347, 2348, 2349, 2350, 2351, 2352, 2353, 2354, 2355, 2356, 2357, 2358, 2359, 2360, 2373, 2374, 2375, 2379, 2380, 2381, 2385, 2386, 2387, 2388, 2389, 2390, 2391, 2392, 2393, 2394, 2395, 2396, 2400, 2401, 2402, 2409, 2410, 2411, 2412, 2413, 2414, 2415, 2416, 2417, 2424, 2425, 2426, 2430, 2431, 2432, 2436, 2437, 2438, 2439, 2440, 2441, 2442, 2443, 2444, 2457, 2458, 2459, 2460, 2461, 2462, 2463, 2464, 2465, 2469, 2470, 2471, 2472, 2473, 2474, 2478, 2479, 2480, 2481, 2482, 2483, 2496, 2497, 2498, 2499, 2500, 2501, 2502, 2503, 2504, 2505, 2506, 2507, 2514, 2515, 2516, 2517, 2518, 2519, 2520, 2521, 2522, 2526, 2527, 2528, 2529, 2530, 2531, 2535, 2536, 2537, 2670, 2671, 2672, 2679, 2680, 2681, 2703, 2704, 2705, 2709, 2710, 2711, 2733, 2734, 2735, 2742, 2743, 2744, 2748, 2749, 2750, 2751, 2752, 2753, 2754, 2755, 2756, 2757, 2758, 2759, 2769, 2770, 2771, 2772, 2773, 2774, 2778, 2779, 2780, 2811, 2812, 2813, 2814, 2815, 2816, 2817, 2818, 2819, 2823, 2824, 2825, 2841, 2842, 2843, 2847, 2848, 2849, 2850, 2851, 2852, 2856, 2857, 2858, 2865, 2866, 2867, 2871, 2872, 2873, 2892, 2893, 2894, 2895, 2896, 2897, 2901, 2902, 2903, 2904, 2905, 2906, 2952, 2953, 2954, 2961, 2962, 2963, 3000, 3001, 3002, 3015, 3016, 3017, 3018, 3019, 3020, 3030, 3031, 3032, 3042, 3043, 3044, 3084, 3085, 3086, 3090, 3091, 3092, 3099, 3100, 3101, 3111, 3112, 3113, 3117, 3118, 3119, 3135, 3136, 3137, 3171, 3172, 3173, 3177, 3178, 3179, 3180, 3181, 3182, 3189, 3190, 3191, 3192, 3193, 3194, 3204, 3205, 3206, 3210, 3211, 3212, 3222, 3223, 3224, 3228, 3229, 3230, 3237, 3238, 3239, 3246, 3247, 3248, 3249, 3250, 3251, 3255, 3256, 3257, 3261, 3262, 3263, 3396, 3397, 3398, 3501, 3502, 3503, 3546, 3547, 3548, 3705, 3706, 3707, 4425, 4426, 4427, 4449, 4450, 4451, 4503, 4504, 4505, 4518, 4519, 4520, 4590, 4591, 4592, 4641, 4642, 4643, 4647, 4648,	Known drug resistance sites in HXB2

		4649, 4656, 4657, 4658, 4668, 4669, 4670, 4671, 4672, 4673, 4692, 4693, 4694, 4722, 4723, 4724, 4782, 4783, 4784, 4974, 4975, 4976, 5016, 5017, 5018, 5067, 5068, 5069, 7863, 7864, 7865, 7866, 7867, 7868, 7869, 7870, 7871, 7872, 7873, 7874, 7875, 7876, 7877, 7881, 7882, 7883, 7884, 7885, 7886	
--min-read-count	Minimum count for each unique read	1	
IQ-TREE			
-m	Substitution model	GTR+F+R6	General Time Reversible rate model, Empirical state frequencies, FreeRate site variation model with six categories
phyloscanner_analyse_trees.R			
splitsRule	Parsimony reconstruction settings	s,15	Sankoff algorithm with *k*=15
--outgroupName	Outgroup name	<HXB2 sequence name>	
--multifurcationThreshold	Multifurcation collapse threshold	1E-5	
--normRefFileName	Per-window branch length normalisation file		Derived from a phylogeny of global HIV diversity constructed using the FreeRate model
--normStandardiseGagPol	Standardise normalising constants so that the average on *gag*+*pol* equals 1		
--parsimonyBlacklistK	Value of *k* in the parsimony algorithm when used to identify contaminant reads	15	
--rawBlacklistThreshold	Threshold of reads below which all tips in a subgraph from a single individual will be blacklisted	10	
--ratioBlacklistThreshold	Threshold for the proportion of all reads from a single individual that are members of a single subgraph, below which all tips from that subgraph will be blacklisted	0·05	
--readCountsMatterOnZeroLengthBranches	Take into account read counts when applying parsimony algorithm		
--distanceThreshold	Distance threshold, normalised by genome position, below which individuals will be identified as a likely transmission pair in a window	0·02	

--allowMultiTrans	Allow the “multiTrans” categorisation to indicate direction of transmission		

Table S5: REGA subtyping results for the individuals involved in the 300 transmission pairs

**Call (source)**	**Call (recipient)**	**Count**
HIV-1 Subtype C	HIV-1 Subtype C	284
HIV-1 Subtype C	Recombinant of C, F1	1
HIV-1 Subtype C, potential recombinant	HIV-1 Subtype C, potential recombinant	1
Recombinant	Recombinant of C, J, H	1
Recombinant of A2, C	Recombinant of C, A1, G	1
Recombinant of C, A1	Recombinant of C, A1	4
Recombinant of C, A1, J	Recombinant of C, A1, J	1
Recombinant of C, A1, J, K	Recombinant of C, A1, J, K	1
Recombinant of C, D, B	Recombinant of C, D	1
Recombinant of C, F1	HIV-1 Subtype C	2
Recombinant of C, G	Recombinant of C, G	1
Recombinant of C, J	Recombinant of C, J	1
Recombinant of C, K	Recombinant of C, D, F1	1

References

1 Fogel JM, Bonsall D, Cummings V, *et al.* Performance of a high-throughput next-generation sequencing method for analysis of HIV drug resistance and viral load. *J Antimicrob Chemother* 2020; **75**: 3510–6.

2 Jenkins F, Le T, Farhat R, *et al.* Validation of a HIV whole genome sequencing method for HIV drug resistance testing in an Australian clinical microbiology laboratory. medRxiv. 2023; published online July 6. DOI:10.1101/2023.07.05.23292232.

3 Minh BQ, Schmidt HA, Chernomor O, *et al.* IQ-TREE 2: New Models and Efficient Methods for Phylogenetic Inference in the Genomic Era. *Mol Biol Evol* 2020; **37**: 1530–4.

4 Nguyen L-T, Schmidt HA, von Haeseler A, Minh BQ. IQ-TREE: a fast and effective stochastic algorithm for estimating maximum-likelihood phylogenies. *Mol Biol Evol* 2015; **32**: 268–74.

5 Ratmann O, Grabowski MK, Hall M, *et al.* Inferring HIV-1 transmission networks and sources of epidemic spread in Africa with deep-sequence phylogenetic analysis. *Nat Commun* 2019; **10**: 1411.

6 The HIV Prevention Trials Network. https://www.hptn.org/sites/default/files/inline-files/HPTN%20071-2%2C%20Version%202.0%20%2807-14-2017%29.pdf (accessed Nov 23, 2021).

7 Liu TF, Shafer RW. Web resources for HIV type 1 genotypic-resistance test interpretation. *Clin Infect Dis* 2006; **42**: 1608–18.
